# Co-creation of self-management support during inpatient stroke rehabilitation

**DOI:** 10.1016/j.pecinn.2023.100191

**Published:** 2023-07-10

**Authors:** Joshua Dobe, Louise Gustafsson, Kim Walder, Kylie Bower, Rosa Lachman

**Affiliations:** a Discipline of Occupational Therapy, School of Health Sciences and Social Work, Griffith University, Queensland, Australia; b The Hopkins Centre, Menzies Health Institute of Queensland, Griffith University, Queensland, Australia; c Occupational Therapy Department, Princess Alexandra Hospital, Brisbane, Australia; d Griffith University, Queensland, Australia

**Keywords:** Cerebrovascular accident, Participatory, Co-design, Co-production, Neurological rehabilitation, Consumer engagement, Hospital

## Abstract

**Objective:**

This study aimed to identify priority self-management skills and behaviours in partnership with stroke survivors, and to co-create approaches to support self-management during inpatient stroke rehabilitation.

**Methods:**

Three stroke survivors and two communication partners participated in the three-stage Participatory Action Research project with embedded co-design processes after undertaking inpatient rehabilitation at a metropolitan tertiary hospital.

**Results:**

Participants identified key factors influencing self-management during inpatient rehabilitation including motivation, emotional well-being, and fatigue. Three approaches to support people to self-manage post-stroke were co-created. (1) A health professional concierge and early family meeting. (2) A peer support person. (3) Adapting the hospital environment.

**Conclusion:**

Findings suggest post-stroke self-management support should commence during inpatient rehabilitation to optimise its research-informed benefits. This support should focus on empowering stroke survivors and their key support people through active involvement in decision-making, and provision of multi-modal individualised education. The impact of hospital environments on emotional-wellbeing and self-management post-stroke also requires further investigation.

**Innovation:**

The identification of a health professional concierge as a co-designed solution to the current challenges with self-management support is an innovative recommendation for practice. The findings support changes to the traditional processes of rehabilitation towards a consumer and family-led practices.

## Highlights

• A foundation of self-management support is required during inpatient stroke rehabilitation
• Co-creation approaches are yet to be commonly used for the development of inpatient stroke rehabilitation practices.
• Self-management support for stroke survivors during stroke rehabilitation requires individualised education and a family-centred approach.
• Active, early involvement in should be prominent during inpatient stroke rehabilitation.

## 1 Introduction

Stroke is a leading cause of disability, with people experiencing deleterious impacts on life roles and their ability to live independently and engage meaningfully in the community [[1], [2], [3], [4]]. Self-management is defined as all the people do to manage their recovery, rehabilitation, health, and everyday life after stroke [4,7]. Self-management behaviours can support adjustment to changing life roles, and the physical and emotional impacts of stroke thereby enhancing health outcomes and quality of life [[4], [5], [6], [7], [8]]. The development of self-management skills such as problem-solving, decision-making, resource utilisation, taking action, and developing partnerships with health professionals are essential precursors to self-management behaviours [4,9].

Support approaches to effectively develop self-management skills after stroke are yet to be conclusively identified and hospital-based self-management support approaches are currently underexplored [5,6,[10], [11], [12], [13]]. Greenway et al. [10] identified hospital-based self-management lacked conceptual clarity and was inconsistently applied between health professions and across the stroke continuum of care. Despite a systematic review highlighting the necessity of including key stakeholder perspectives in self-management support development [14], limited research exists incorporating stroke survivor perspectives [2,3]. With contemporary health care services championing principles of consumer engagement and patient-centred care [15], an opportunity exists to privilege stroke survivor perspectives to develop effective self-management support during inpatient rehabilitation.

Stroke survivors experience frustration and disempowerment during inpatient rehabilitation [16], and their lived experience and perspective can inform development of effective self-management support in this context. Co-creation is an approach incorporating stakeholder perspectives in healthcare service development [17]. Described as the “zeitgeist” of our times in quality improvement, co-creation partners consumers of the service with researchers to co-design healthcare services [[18], [19], [20]]. Co-creation in the hospital context is associated with increased acceptability, uptake, and usability of interventions and services [21]. Ultimately, co-creation is a service improvement methodology that develops person-centred interventions, facilitating greater health impact, and improved health outcomes [17,22,23]. The purpose of this study was to employ co-creation to identify priority self-management skills and behaviours in partnership with stroke survivors, and develop self-management support during inpatient stroke rehabilitation.

## 2 Methods

**Fig. 1:**
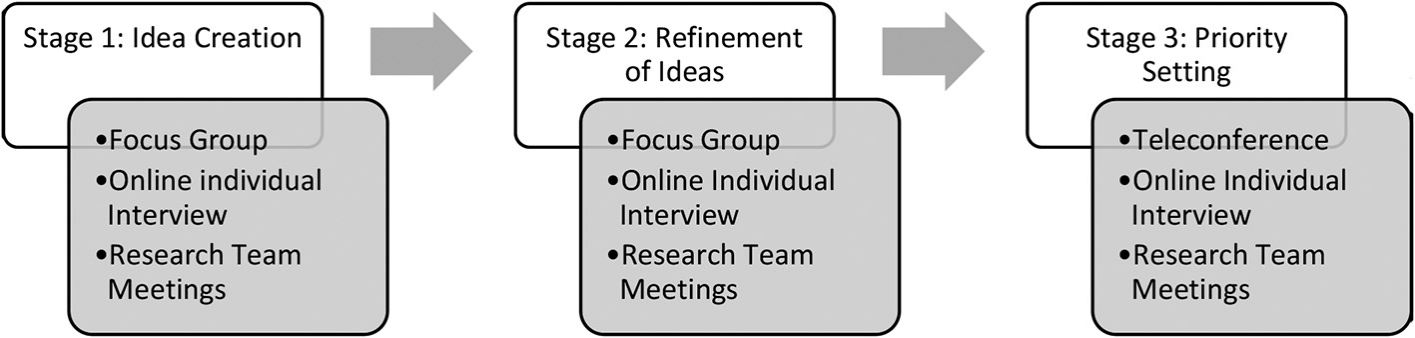
Co-Creation Process.

A three-stage Participatory Action Research (PAR) approach adapted from Ogrin et al. [22] (Fig. 1) guided the study, underpinned by the pragmatic research paradigm [24]. A key principle of co-creation and PAR is involvement of key stakeholders through the research process; thus, the research team included a stroke survivor and clinician researcher who were involved in all stages of the research process.

### 2.1 Ethics

Ethical approval was granted from Metro South Human Research Ethics Committee [HREC/2021/QMS/70512] and Griffith University Research Ethics Committee [2021:194]. All participants received written and verbal information regarding the study purpose and procedures, and possible risks and benefits. Formal written consent was gained from all participants and verbal consent was gained at the commencement of each data collection event.

### 2.2 Study context

All potential participants received inpatient rehabilitation on a general rehabilitation ward for people with stroke and a range of other health conditions. The interprofessional team included dieticians, doctors, nurses, occupational therapists, pharmacists, physiotherapists, social workers, and speech pathologists with limited psychology or neuropsychology services available. People discharged from the inpatient unit are referred to an onsite outpatient day hospital or community-based transition care or rehabilitation services for continuing rehabilitation.

### 2.3 Participants

Convenience sampling aimed to recruit 6–8 stroke survivors discharged from the inpatient rehabilitation unit and/or accessing the outpatient day hospital clinic. The inclusion criteria were: diagnosis of stroke; ≥18 years of age; living in the community; ability to provide informed consent and participate in the qualitative data collection (with or without communication partners); no co-morbidities significantly altering stroke rehabilitation i.e., diagnosis of advanced dementia, severe mood disorder or chronic mental health condition.

### 2.4 Procedure

**Fig. 2:**
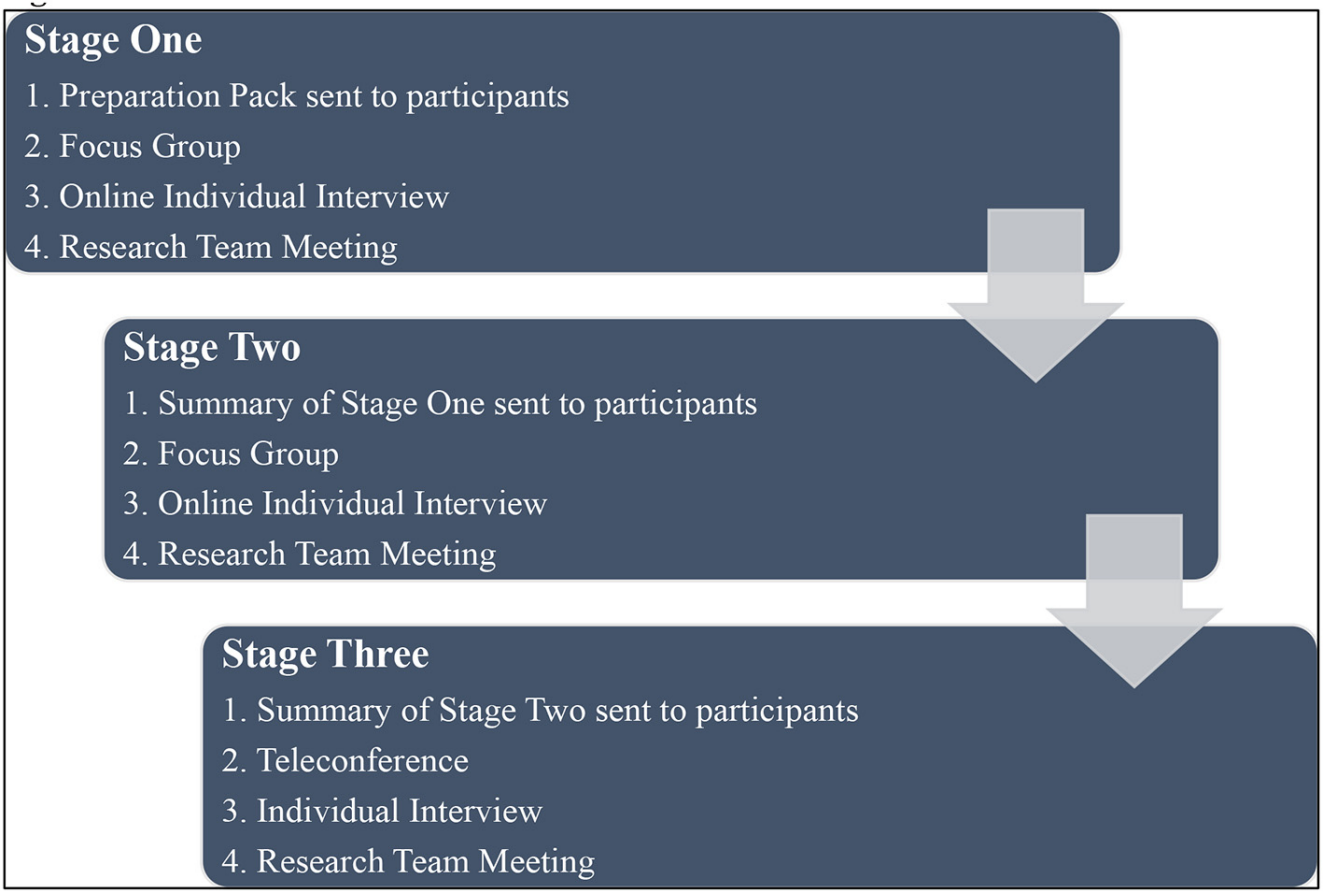
Data Collection Procedure.

Participants were involved in a series of three co-creation stages (Fig. 2). Each co-creation stage (data collection event) was facilitated by JD and LG and ranged in duration from 45 to 120 min. To promote inclusivity, participants were able to attend a focus group or an individual interview. Consistent with PAR, each data collection event was followed by research team meetings to plan for the subsequent event (Fig. 2).

#### 2.4.1 Idea creation

The purpose of Stage One was to identify the priority issues post-stroke that could be addressed through self-management and propose potential approaches to support this skill and behaviour development. All participants received an information pack that introduced self-management prior to this stage. At the data collection event, participants and researchers introduced themselves, the aims and expectations of the research were clarified, and a shared understanding of self-management was established. Discussions were guided by open-ended and probing questions from the question guide (see supplementary material) and the direction of the discussions. The discussions were audio-recorded and transcribed, and field notes were taken by the researchers.

#### 2.4.2 Refinement of ideas

Participants were emailed preparatory information including key issues identified for self-management support, service approaches and the aim of Stage Two. This information was reviewed at the beginning of the discussions to ensure they were an accurate reflection of participant perspectives. Guided discussions supported participants to further refine and develop these approaches including consideration of the timing of support, operationalisation of service provision, and pragmatics of the approaches in the inpatient rehabilitation environment. The discussions were audio-recorded and transcribed, and field notes were taken by the researchers.

#### 2.4.3 Priority setting

Participants received a summary of the outcomes from Stage Two in preparation for the discussions to finalise and prioritise the approaches. Participants collaborated to make decisions regarding the importance of each approach and which approaches should be pursued further. Consensus was achieved through the discussions, which were audio-recorded, and field notes were taken by the researchers.

### 2.5 Data analysis

The data analysis was iterative in nature, using the knowledge created to inform action and the next stage of co-creation [25]. Data analysis was conducted concurrently and immediately following data collection utilising a combination of formal and informal analysis processes [25]. Two researchers (JD, LG) conducted the initial, informal data analysis with discussions immediately following the data collection events, incorporating field notes and recollections of the discussions. Preliminary codes were identified but were not confirmed until formal analysis was completed. This informal analysis continued with the summary of the discussions and preliminary codes presented and discussed at the research team meeting – research team members were able to listen to the recorded focus group prior to this meeting -and this supported the clinician and lived expert researchers to contribute to the interpretations. A final, formal analysis process was then completed with the transcripts and involved line by line content analysis [26,27]. The content analysis [26] supported the confirmation or refinement of the preliminary codes and the emergence of new codes. This process was adopted for pragmatic reasons due to COVID delays and the transcription timeframes and included constant reference back to the recordings and transcription to ensure that the participant voice was prioritised at all times.

### 2.6 Trustworthiness

The five criteria for qualitative methodological trustworthiness, credibility, transferability, dependability, confirmability, and authenticity, were considered through this study's design and implementation [28,29]. The researchers facilitating data collection events and data analysis (JD,LG) enacted a process of reflexivity through use of research journals. Assumptions, perspectives, and reflections recorded in these journals were discussed openly during research team meetings. Member checking was conducted when the summaries of findings were presented and discussed at the subsequent data collection event. Transferability was supported through a detailed description of research methods utilised. LG, KW and KB are experienced stroke rehabilitation researchers and the engagement with participants occurred over a prolonged two-month period. Direct quotes were used to report results and findings are linked to previous research to promote authenticity and confirmability.

## 3 Results

### 3.1 Participants

Seven stroke survivors were invited to participate and three stroke survivors with two communication partners agreed. One stroke survivor was unable to be contacted (*n* = 1), one was unable to commit to the data collection (*n* = 1) and two were unwilling with no reasons specified (*n* = 2). Three male stroke survivors participated and their pseudonyms and demographic details are outlined in Table 1.

**Table 1:** Participant Demographic Information.

Participant	Age	Marital Status	Time in Hospital Post-Stroke (Months)	Time Post-Stroke (Months)	Most Significant Self-Reported Changes Post Stroke
Nathan Communication Partner: Christine	69	Married/Defacto	4.5	18	“Dizziness” “Headaches” “Sleepy” “Can't walk” “Bad Speech”
Thomas Communication Partner: Eliza	61	Married/Defacto	5.5	7	“Non-verbal” “Paralysis” “Temperature regulation – always cold” “Learn to swallow and eat” “Child-like response to situations”
Nicholas	46	Married/Defacto	1	6	“Always tired” “Short Temper” “Right-side weakness” “Can't relay how I am feeling effectively” “Can't type”

Two stroke survivors participated in the focus groups with communication partners and they were considered as dyads. Dyad one were: Thomas who had severe expressive aphasia and Eliza (spouse and communication partner) who spoke in the focus groups. Thomas was involved through yes/no questioning that allowed him to answer closed questions or agree/disagree with what had been conveyed by Eliza, Dyad two were Nathan who had a cognitive communication disorder and Christine (spouse and communication partner). Nathan would respond to questions and Christine would provide clarification or detail – as a result included quotes are often Christine representing what Nathan was wishing to convey. The third stroke survivor (Nicholas) was unable to attend the focus group due to work commitments and participated in an online individual interview, which always followed the focus group.

### 3.2 Idea creation

Participants identified sequelae of stroke that were challenging to self-manage, barriers to self-management during inpatient rehabilitation, and unmet needs that could be addressed through self-management. These included motivation, fatigue, emotional well-being, family engagement, individualised early education, and health professional relationships. Potential approaches to support this skill and behaviour development were introduced.

#### 3.2.1 “There's no shortcut”

Motivation enabled or inhibited engagement in ongoing therapy, re-engagement with previous roles, and self-management. Motivation was difficult in the early stages of rehabilitation with Nathan preferring “to have my food in the bed” while the nurses “tried to get me to go into the dining room”. Christine added that the low motivation extended “Even down to the point where they [nurses] would say, right, you know it's time to go and have a shower, and he'd say, “No, not having a shower. No.” The turning point for Nathan was a meeting with a peer support person:

I looked at this bloke, and I thought, I see what he's been through, you know, I'm not that bad. You know, get me finger out and, do something about, you know. After that, …..that's when I started to go into the dining room and, then start becoming pretty good after that.

The challenges with motivation heightened on return to home without the structure of rehabilitation and impacted community re-engagement:

It's like yes, you've got your other half there pushing you to do it so that you're going to get better and you're going to improve everyday … but when you're at home, it's like the wife's only nagging … I don't want to do it because you know, just leave me alone. (Eliza)

#### 3.2.2 “If you're tired, you're tired”

Participants highlighted the difficulties of managing fatigue. Nicholas described that “usually, it takes a couple of hours and then I just feel tired and want to go off [to sleep] and brain sort of goes into neutral as well”. Christine explained that she and Nathan had accepted that “You've got to work your way through it because you just don't know when the fatigue is going to be there, how long it is going to be there for.” The difficulties of managing this fatigue during occupational engagement were expressed, “On the weekend when I'm out walking around with my kids, I can't rest. I can't rest in the middle of a golf game.” (Nicholas) Strategies to self-manage fatigue ranged from the recognition that “If I'm tired, I know I have to sit down and have a break.” (Nicholas), through to Eliza who described a structured approach to managing Thomas's fatigue and therapy:

We had sort of a roster set up because of fatigue and things like that … We had to have, he could do, have physio for an hour and then we had to have an hour break, and then we'd have speech and then he'd have a rest, and then lunch, and then ….

#### 3.2.3 “Confronting”

It was believed that emotional well-being was a crucial factor that limited the stroke survivors' capacity to self-manage. Emotional well-being was a significant factor from “the start of emergency all the way through [their hospital stay]” and was compounded by the hospital environment that is “just so foreign and confronting”. The transition to rehabilitation was described as entering “a whole new world” and Eliza stated,

I know it's very busy, but it needs a little more humanisation… You've lost a lot of dignity. So, it's those hurdles as well as the emotional stuff that you've got to get past, I think, to even start to develop the concept that you have to do the therapies to get out.

The impact of emotional well-being on self-management skills was highlighted. “I didn't have any goals to be started with because ….. I was down on myself. Even now I'm still down.” (Nicholas) Participants identified the need for additional support for emotional well-being “Especially if you're talking about the mental state, you need assistance, like the psychologist talking to them, rather than giving them a pill.” (Eliza) This was considered important to prepare for the transition to home, “Because I think if there's more the mental health [support] and that assistance at the beginning, by the time we get them home and have to deal with it, it's more an acceptance.” (Eliza)

#### 3.2.4 “Being part of the process”

The active involvement of the stroke survivor and key support people in therapy and decision-making would facilitate development of self-management skills. Eliza stated, “I think it would help their [stroke survivor] recovery because they're part of the process” further explaining that at the moment “The patient doesn't seem to be part of the process.” As an extension to this, the spouse was also not often included:

I was not going to rehabilitation in the morning, because obviously that was peak therapy time, and … they don't encourage you to be there in those times. Which is fine to a degree. But I think for me personally, I missed out on a lot of learning. (Christine)

Eliza explained this concept of active involvement in decision-making and therapy as essential for self-management:

I think that's something that needs to be part of self-management but right from the beginning. Everything seems to be, let's wait until we get home to self-manage. We need to do it from here. If we're going to recover, we want it here as we're doing our recovery. We need to start self-management here.

#### 3.2.5 “Buy-in at the beginning, not the end”

Participants described the opportunity to facilitate self-management through “humanisation” of the education, “You know, that someone just to sit down and talk to you and run through [rehabilitation process].” (Eliza) The value of early and individualised education was considered important in contrast to what was received “The education that is given is very broad…But I think if there was something that was a little more targeted for different types of stroke.” (Christine). The incorporation of a family meeting early in the rehabilitation process to facilitate this active involvement of stakeholders and delivery of individualised education was discussed.

A session to tell you what's going on, like we're all your therapists, we're going to do work to do blah with you, to get you to a stage to be able to go home and function…a family meeting sort of thing at the beginning…have other family members who are going to support you come along as well…So that you've got the buy-in by everyone else. That buy-in at the beginning, not at the end. (Eliza)

#### 3.2.6 “The greatest impact”

The positive influence of relationships with members of the healthcare team during the inpatient rehabilitation journey was noted. Participants discussed the perceived benefits of having familiar staff to approach if required. “The nurses, you get your team of nurses, and he likes routine and the familiarity.” (Eliza). Christine explained the importance of the first encounter with health care staff and the overall influence the interaction can have on the stroke survivor and family well-being:

What I found was, your first nurse that you come in contact with is the one that has the greatest impact. She said to my son and I as a family, she said, “Don't be negative. It's going to be okay. Just keep the positive. All the good.”

### 3.3 Idea refinement

The findings for stage one were discussed and informed further discussions of approaches to self-management support. The focus was on building a “foundation” with early and active involvement to develop self-management skills more effectively. The suggested approaches included a health professional concierge, family meeting, peer support, adapting the hospital environment, and implementing individualised education. These are illustrated in the themes below.

#### 3.3.1 “The foundation”

The importance of active involvement of the stroke survivor and key supports to facilitate self-management was confirmed. Participants detailed the need to address emotional well-being to catalyse motivation, “You don't get the motivation if you don't have the emotional well-being.” (Christine) Nicholas explained “I would have loved to plan my therapy session a little bit”. The impact of individualised education to actively involve people in the therapy was stressed “He had no great understanding why he was doing a particular task. And if it didn't interest him then motivation was low.” (Christine). Participants described the need for stroke survivors and supports to “become part of that journey, to help the therapists and everybody else.” (Eliza). Education and information were a motivator for Nicholas who explained that information about the importance of early therapy intervention encouraged him to take an active role in therapy and self-manage, “I did that [exercises] because I wanted to get better, and because I knew for a fact that the first six months … that's when you can become better.” However, Nicholas reinforced that “It's [education] got to be tailored to each specific need… I would tailor it [education] individually instead of just a blanket approach.”

It was stressed that addressing emotional well-being, implementing individualised education, and supporting active involvement would build the foundation upon which the motivation and acquisition of self-management skills and behaviours would occur.

Because once you know what you're looking for, you'll be motivated for the end goal. See, when you don't know, what have you got to look forward to, really? There's nothing to motivate you, because, yeah, you've got to get home, but how are you going to get home? (Eliza)

#### 3.3.2 “Right from the start”

The opportunity to build this “foundation” started with the initial transition to rehabilitation. Participants introduced the idea of a “concierge”, someone who would meet with the person on admission to rehabilitation and guide them with individualised information about their stroke, the processes of rehabilitation, and what to expect. The importance of an early family meeting was reinforced:

I think the whole thing [transition to rehabilitation] probably needs like, some guidelines, with everything, you know? … when they go in the stroke ward, it's explained to them what's going to happen and where you've got to go to rehab…have a family meeting, explain to everybody so they're all on the same page, know what's going on. (Eliza)

The negative impact of the hospital environment on emotional well-being was identified as a barrier to this “foundation”. Adapting the physical and cultural environments of rehabilitation was encouraged:

I just found the rooms were really depressing to be in … there was no lightness in the rooms. Like, I don't know how else to describe it as lightness, it is business, and it has to be business. (Christine)

It needs to be a) let's encourage families in, let's encourage people to sessions, let's encourage family to bring pictures and paintings and things like that. Let's encourage family to bring food. (Eliza)

Finally, meeting a peer support person early in rehabilitation to deliver individualised education, support emotional-well-being, and enable stroke survivor empowerment was suggested:

Because I think if you can look at a person who's been through what [stroke survivors] have been through and then come out to a degree … maybe that interpersonal reaction would help to say well, you know, I've been at a bad place. But I was able to work through it and now I can do XYZ. (Christine)

### 3.4 Priority setting

Three approaches were refined, prioritised, and finalised during Stage Three: (1) A health professional concierge and early family meeting (incorporating individualised education); (2) A peer support person; (3) Adapting the hospital environment.

**Fig. 3:**
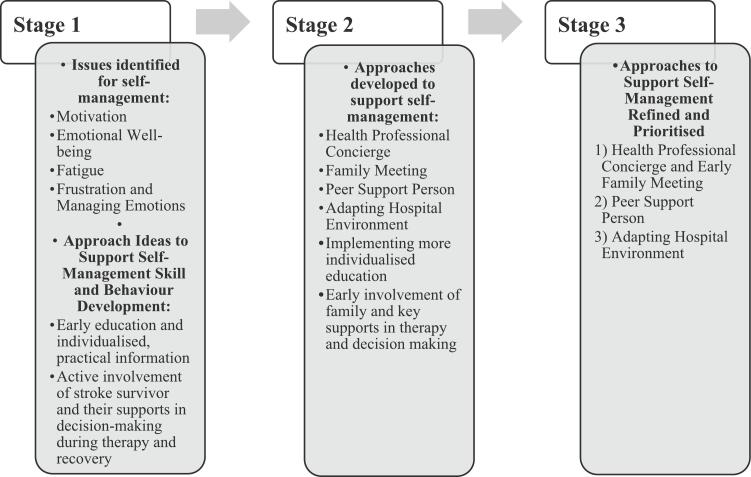
Summary of Findings.

These prioritised approaches are detailed in Table 2. A summary of the findings of each stage are displayed in Fig. 3.

**Table 2:** Approaches Developed to Support Self-management During Inpatient Rehabilitation.

Approaches	Details
1. Health Professional Concierge and Early Family Meeting	Health Professional Concierge: Member of health care team to provide: - Early education upon arrival to rehabilitation which includes: - What is rehabilitation? - What is expected of the stroke survivor and family during their stay in rehabilitation? - Information on pathway from admission to discharge. - How the stroke survivor and family can be involved in therapy. o This would include a tool or resource to support goal setting and action planning. Early Family Meeting: - Provide opportunity to share information and ask questions. - Include individualised education - Education on how the stroke survivor and their family can be involved in therapy. - Information on pathway from admission to discharge.
2. Peer Support Person	Peer Support Person: - Involve introductory groups sessions and subsequent weekly individual sessions. - Provide an opportunity for peer support person to discuss their recovery and stroke survivors to discuss concerns, difficulties, and well-being.
3. Adapting Hospital Environment	Implement changes to the inpatient rehabilitation environment to support motivation, empowerment and individual control over environment: - Control over environment: family photos, flowers, pictures etc.

## 4 Discussion and conclusion

### 4.1 Discussion

This PAR study sought to identify priority issues and develop approaches that support self-management skill and behaviour development during inpatient stroke rehabilitation. Through the operationalisation of co-creation, the participants identified that a “foundation” of active involvement, individualised education, and support for emotional well-being was required. Participants detailed three priority approaches to achieve this: a health professional concierge and early family meeting, peer support person, and adapting the hospital environment. This “foundation” of self-management support is consistent with the concept as described by Parke et al. [4]. The findings challenge the current rationalisation occurring within healthcare settings due to shrinking funds and resources and emphasise the importance of individualised and family-centred approaches. Innovative approaches to self-management support during inpatient rehabilitation have been proposed that could address the identified need to understand the optimal timing and the key features of effective self-management support [4,7,[10], [11], [12], [13]].

Stroke survivors experience negative emotions during inpatient rehabilitation including disempowerment, frustration, feelings of lost control of their situation and environment, and isolation [16,30]. Self-efficacy, a concept closely aligned to an individual sense of empowerment, is the mediator which enables or inhibits self-management behaviours [4,7,14,31]. Our participants believed that the research informed benefits of self-management can be optimised if the environment of inpatient rehabilitation is enhanced to promote stroke survivor empowerment and emotional wellbeing. In the context of limited and primarily exploratory research investigating the impact of the hospital environment on stroke survivor emotional well-being and empowerment [16,32], further research is required to evaluate this relationship and impact on self-management outcomes.

It is well established that there is a need for active and patient-specific information provision [4,5,8,33] to support active engagement in rehabilitation [34]. In 2011, Maasland et al. suggested that stroke survivor knowledge of their conditions was commonly suboptimal [35]. Group-based education and generic written materials have been commonly instigated to overcome this issue. Recently, Clark et al. identified group self-management should be personalised and tailored to the individual to increase motivation to engage in the program [2]. Equally, an evaluation of a post-stroke six-month review process identified that the uniform approach limited the benefits and a patient-led, individualised approach to education and self-management should be considered [36]. Individualised education and adequate information provision significantly contribute to stroke survivor empowerment [30,34,37] and our findings highlight that stroke survivors value individualised and multi-modal education to support development of self-management skills and behaviours.

A multi-modal approach to information provision harnesses the benefits of both peer supports and health professionals. Peer support positively influences self-efficacy and emotional well-being post-stroke [2,8,[38], [39], [40], [41], [42]] and is a possible mediator of effectiveness of self-management interventions [8]. However, peer support workers can have negative influences if there is no access to training and support. A peer support worker role is well established within mental health practice and could be considered as a standard for translation into stroke rehabilitation. Similarly, forming partnerships with health professionals is established as a key self-management skill [4]. The Health Professional Concierge approach proposed in this study aims to achieve this from the initial stages of inpatient rehabilitation. Rehabilitation therapies, goal setting and action planning guided by health professional support, and structured information provision will enable self-management [4,8,33]. There is an opportunity for health services to consider a dual approach of peer support and a health professional concierge to enhance self-management support and outcomes.

Finally, the role of family, supports and carers in self-management post-stroke were highlighted. Family, carers, and support people face significant challenges and are intimately involved in supporting stroke survivors to adjust to the sudden life altering impacts of stroke [41,[43], [44], [45]]. Self-management support incorporated during inpatient rehabilitation should consider, involve, and acknowledge the pivotal role of family and key supports [2,8,33]. Indeed, Satink et al. concluded that spouses of stroke survivors should be included as full participants in self-management interventions [41]. Considering both the stroke survivor and their key support as a dyad when implementing self-management approaches could support the development of relevant self-management behaviours. There is a need for this study to be replicated across different settings to understand if the recommendations are generalisable.

This study was limited to participants from one inpatient rehabilitation unit, which supported the development of targeted recommendations, but a larger sample size from more services may yield different results. The COVID-19 pandemic may have influenced the participants rehabilitation experience and impacted the study, with the final data collection event occurring entirely online or via telephone. However, due to the prolonged engagement with participants and the relatively small sample size supporting increased rapport between participants and researchers, it is not considered that this significantly influenced data collection. The small sample size could be considered a limitation however the three participants were representative of the heterogeneous male stroke survivor population due to the range of stroke impacts that they experienced. It is unclear if the findings would have been different if female stroke survivors had been involved. The inclusion of two participants who required communication support partners is a strength of the study but may be considered to limit the findings to the perspectives of spouses. The data collection process was conducted to ensure that the discussions were representative of the stroke survivor and their experience. Finally, due to pragmatic reasons including COVID delays and transcription timelines, the informal analysis and discussion of the data collection episodes occurred prior to receipt of the transcript. Although we do not believe that this occurred, it may be considered that this process limited the prioritisation of the participants' voices during the analysis process.

### 4.2 Innovation

Co-design of approaches for inpatient stroke rehabilitation is a novel field, with previous work predominantly for community- based context and frequently related to development of technology-based interventions [46]. Privileging the experiences and expertise of stroke survivors and their key support people through this co-design process challenges the expert-driven medical model that is prominent within traditional practices of inpatient rehabilitation.

The findings that link the introduction of a health professional concierge as a foundational requirement for self-management suggests an innovative solution for inpatient rehabilitation that has not been previously evaluated with respect to the development of self-management skills and behaviours.

## 5 Conclusion

An evidence base has rapidly developed over the last decade, identifying the benefits of stroke self-management but concluding that the optimal delivery and timing remain unrecognised. The findings suggest that the impact of integrating self-management support from the beginning of inpatient rehabilitation should be further investigated. It is proposed that to realise the benefits of self-management, stroke survivors must be firstly empowered, actively involved in therapy and emotionally supported through the provision of multi-modal individualised education, peer support, and carer involvement during inpatient rehabilitation. Furthermore, the rehabilitation environment must be adapted to support stroke survivor empowerment and emotional well-being to foster motivation, self-management skills and self-efficacy. Without this shift in rehabilitation environments and without building a “foundation” to support self-management, stroke survivors are not being optimally supported to manage the sudden, life-changing, and chronic impacts of their condition.

## Funding

Study supported by 10.13039/501100001791Griffith University Honours Funds.

## CRediT authorship contribution statement

**Joshua Dobe:** Conceptualization, Methodology, Formal analysis, Investigation, Writing – original draft, Visualization, Project administration. **Louise Gustafsson:** Conceptualization, Methodology, Formal analysis, Investigation, Writing – review & editing, Visualization, Supervision, Project administration. **Kim Walder:** Conceptualization, Methodology, Formal analysis, Writing – review & editing, Supervision. **Kylie Bower:** Conceptualization, Methodology, Formal analysis, Writing – review & editing. **Rosa Lachman:** Conceptualization, Formal analysis, Writing – review & editing.

## Declaration of Competing Interest

None.
